# Epidemiologic evaluation of human papillomavirus type competition in unvaccinated women from Xiamen, China

**DOI:** 10.3389/fmicb.2025.1546166

**Published:** 2025-04-04

**Authors:** Yu Chen, Qing Li, Liya Du, Zhuowen Du, Yixi Zhou, Yanru Huang, Jian Zhang, Wenbo Wang, Lutan Zhang, Jieqiong Xie, Chao Xu, Yunsheng Ge, Xingmei Yao, Yulin Zhou

**Affiliations:** ^1^Department of Central Laboratory, Fujian Key Clinical Specialty of Laboratory Medicine, Department of Obstetrics and Gynecology, Women and Children's Hospital, School of Medicine, Xiamen University, Xiamen, Fujian, China; ^2^United Diagnostic and Research Center for Clinical Genetics, Women and Children's Hospital, School of Medicine and School of Public Health, Xiamen University, Xiamen, Fujian, China; ^3^Women's Health Care Department, Department of Obstetrics and Gynecology, Women and Children's Hospital, School of Medicine, Xiamen University, Xiamen, Fujian, China; ^4^Department of Quality Control, Department of Obstetrics and Gynecology, Women and Children's Hospital, School of Medicine, Xiamen University, Xiamen, Fujian, China; ^5^Department of Information Center, Department of Obstetrics and Gynecology, Women and Children's Hospital, School of Medicine, Xiamen University, Xiamen, Fujian, China

**Keywords:** human papillomavirus (HPV), infection, type competition, cervical cancer, HPV vaccine

## Abstract

**Background:**

Human papillomavirus (HPV) vaccination is expected to reduce the burden of cervical cancer and other HPV-related diseases. However, if competition exists among HPV types, type replacement may occur following the reduction of vaccine-targeted types. Here, we conducted the study to explore natural HPV type competition in unvaccinated women.

**Methods:**

HPV DNA test results from cervical samples collected between January 2013 and July 2023 at Xiamen University's Women and Children's Hospital were analyzed. In cross-sectional study, first-visit HPV genotyping results were used, and logistic regression model was constructed to evaluate interactions between vaccine-targeted and other HPV types. In cohort of women with multiple visits, the risk of acquiring other HPV types was compared between women infected with vaccine-targeted types and those HPV-negative using Cox proportional hazards model.

**Results:**

Among 159,049 women, 19.8% tested HPV-positive, with 5.1% having multiple types. Significant negative associations were observed between HPV-6 and HPV-72 (OR: < 0.01; 95%CI: < 0.01–0.03), HPV-18 and HPV-72 (OR: < 0.01; 95%CI: < 0.01–0.02), HPV-31 and HPV-83 (OR: < 0.01; 95%CI: < 0.01–0.55), HPV-33 and HPV-26 (OR: < 0.01; 95%CI: < 0.01–0.81), HPV-45 and HPV-55 (OR: < 0.01; 95%CI: < 0.01– < 0.01), HPV-56 and HPV-26 (OR: < 0.01; 95%CI: < 0.01–0.09), as well as HPV-59 and HPV-69 (OR: < 0.01; 95%CI: < 0.01–0.68), suggesting potential type competition. However, no type competition pair was found in the cohort study. Conversely, women with vaccine-targeted types had a higher risk of acquiring other types (HR > 1.0).

**Conclusions:**

Our findings suggested that HPV-6 and HPV-72, HPV-18 and HPV-72, HPV-31 and HPV-83, HPV-33 and HPV-26, HPV-45 and HPV-55, HPV-56 and HPV-26, HPV-59 and HPV-69 were potential type competition pairs.

## 1 Background

Human papillomavirus (HPV) is a pathogenic microorganism primarily transmitted through sexual contact. Currently, over 200 types have been identified, with more than 40 types associated with genital infections that can be transmitted through sexual contact (Chiarini et al., 2019; Nguyen et al., 2022). Currently, 13 HPV types are classified as high-risk: HPV-16, −18, −31, −33, −35, −39, −45, −51, −52, −56, −58, −59, and−68 (Kreimer et al., 2013). Up to 70% of cervical cancers are attributed to HPV-16 and HPV-18, while collectively, HPV-16, −18, −31, −33, −45, −52, and−58 account for ~90% of cervical cancer cases (Bruni et al., 2021). In recent years, the incidence and mortality rates of cervical cancer in China have shown an upward trend, rising from 3.8/100,000 person-years and 1.2/100,000 person-years in 2003 to 12.3/100,000 person-years and 3.5/100,000 person-years in 2016 (Chen et al., 2013, 2015, 2018a; Di et al., 2015; Zhang et al., 2018; Zheng et al., 2019, 2023). According to the 2023 ICO report (Bruni et al., 2023), in 2020, there were ~110,000 new cases of cervical cancer and about 59,000 deaths in China, accounting for 20% of the global total. Cervical cancer has become one of the significant public health threats to women's health in China.

There are currently six vaccines available worldwide, since the prophylactic vaccine became available in 2006, including the three bivalent vaccines, Cervarix^®^ (GlaxoSmithKline; Wavre, Belgium), Cecolin^®^ (Xiamen Innovax Biotech Co Ltd; Xiamen, Fujian Province, China), and Walrinvax^®^ (Walvax Biotechnology Co; Kunming, Yunnan Province, China) which target the 2 most carcinogenic types, HPV-16 and HPV-18, two quadrivalent vaccines, Gardasil^®^ (Merck & Co, Kenilworth, NJ, USA) and Cervavac^®^ (Serum Institute of India (SII), Pune, India), which target HPV-16, −18, −6, and−11, the later 2 types responsible for ~90% of genital warts (condyloma acuminata), and one nonavalent vaccine Gardasil^®^9 (Merck & Co, Kenilworth, NJ, USA), which targets HPV-6, −11, −16, −18, −31, −33, −45, −52, and−58 (Petrosky et al., 2015; Castle, 2023). The effectiveness of HPV vaccines has been widely proven, and 141 (72.7%) countries have introduced HPV vaccine into their national immunization programs (World Health Organization, 2024). With the implementation of the HPV vaccine immunization program and high coverage, studies have shown a significant reduction in the prevalence of HPV infection and high-grade cervical precancerous lesions (Dong et al., 2021). The domestically developed nonavalent HPV vaccine by Xiamen University has successfully concluded Phase II clinical trials, showing promising immunogenicity, and Phase III clinical trials are currently in progress (Hu et al., 2023).

The preventive HPV vaccine holds the promise of ultimately reducing or even eliminating the burden of cervical cancer and other related diseases caused by HPV infection. However, the vaccine can only prevent infection and lesion related to the vaccine-targeted types, and the current vaccines do not target all high-risk HPV types. It is of great concern whether the “type replacement” would occur after the implementation of HPV vaccination (Man et al., 2019). Type replacement is defined as an increase in the infection rate of certain HPV type due to the elimination of some HPV types (Tota et al., 2017). In previous studies, type replacement has been observed in post-pneumococcal vaccination (Weinberger et al., 2011), raising concerns that a similar phenomenon could occur with HPV vaccine, potentially hindering the success of vaccination in reducing the incidence and mortality of HPV-related diseases. As the HPV vaccination rate will greatly increase with the nationwide promotion of HPV vaccination pilot programs in China, it is crucial to explore the possible of HPV type replacement to evaluate the impact of HPV vaccination on the disease burden of cervical cancer comprehensively, and thereby provide a scientific basis for the formulation of cervical cancer prevention and control strategies in China. The best way to evaluate HPV type replacement is the comparison of infection with non-vaccine-targeted types in vaccinated population vs. unvaccinated population or the comparison of those in post-vaccination vs. pre-vaccination period by long-term surveillance. However, the introduction of HPV vaccine is late and the coverage of HPV vaccine is low in China. It is difficult to directly observe whether HPV type replacement occurs after vaccination. Previous research has proposed that the potential for type replacement after HPV vaccination depends on the competitive interactions between HPV types. Therefore, it is possible to study HPV type competition by evaluating the interactions between HPV types in unvaccinated population (Tota et al., 2013). Recently, Yingying Su et al. explored HPV type competition in an unvaccinated population, and the results suggested the possibility of type competition between HPV-16 and HPV-52, HPV-18 and HPV-51, −52, −58, HPV-31 and HPV-39, −51, −52, −53, −54, −58, HPV-33 and HPV-52, −58, HPV-58 and HPV-52, HPV-6 and HPV-39, −51, −52, −53, −54, −56, −58. However, this study was based on cross-sectional data with a limited sample size (Su et al., 2024). In this study, we plan to evaluate the interactions between HPV types through cross-sectional and cohort studies with a larger sample size to assess the potential for HPV type competition and type replacement.

## 2 Methods

### 2.1 Subject population and specimen collection

The subjects in the study were women who underwent HPV testing at the Women and Children's Hospital, School of Medicine, Xiamen University, China from January 2013 to July 2023. Participants lacking age and identity information were excluded from the study. Gynecologists collected cervical exfoliated cells from outpatient women strictly following standard procedures, which were then preserved in a solution for HPV DNA analysis. The study was approved by the Ethics Committee of Women and Child's Hospital, School of Medicine, Xiamen University (approval number KY-2024-025-K02).

### 2.2 HPV DNA testing

Two commercial HPV GenoArray Diagnostic kits, HBGA-21PKG and HBGA-37PKG, were used for HPV DNA typing, detecting 21 and 37 types, respectively. The HBGA-21PKG kit (2013-2017) identified 13 HR-HPV (HPV-16, −18, −31, −33, −35, −39, −45, −51, −52, −56, −58, −59, and−68) and 8 LR-HPV (HPV-6, −11, −42, −43, −44, −53, −66, and−81) types, while the upgraded HBGA-37PKG kit (since 2018) can detect other 16 types (HPV-26, −34, −40, −54, −55, −57, −61, −67, −69, −70, −71, −72, −73, −82, −83, −84). By using polymerase chain reaction (PCR) to amplify HPV DNA extracted from cervical samples, the resulting amplicons are hybridized with specific HPV probes within the patented “HybriMem” system, utilizing our US-patented flow-through hybridization technology. The results are obtained through a colorimetric detection method using enzyme immunoassay. Both kits were approved by the National Medical Products Administration (NMPA) and have high sensitivity and specificity (>95%) compared to FDA approved HPV Genotyping assay (Liu et al., 2010; Yang et al., 2016). They are widely recognized in medical institutions and scientific research (Hybribio, 2012; Baloch et al., 2016; Li et al., 2016; Luo et al., 2020; Gao et al., 2021). The tests were performed according to the manufacturer's protocol, which has been detailed in our previous publication (Yao et al., 2024). Briefly, the experimental protocol includes DNA extraction using magnetic beads, PCR amplification, hybridization using the HPV GenoArray Diagnostic Kit, and signal processing. Extracted DNA was subjected to PCR amplification using HPV L1 consensus primers, followed by flow through hybridization on a probed membrane for the detection of HPV types. The results were manually interpreted using the provided guide, the blue dot on the probed membrane indicated a positive result, multiple dots showed multiple infections. Controls were included for quality assurance in each test (Ajenifuja et al., 2018; Wang et al., 2018; Wüstenhagen et al., 2018; Shen et al., 2023; Yao et al., 2024).

### 2.3 Statistical analysis

**Figure 1:**
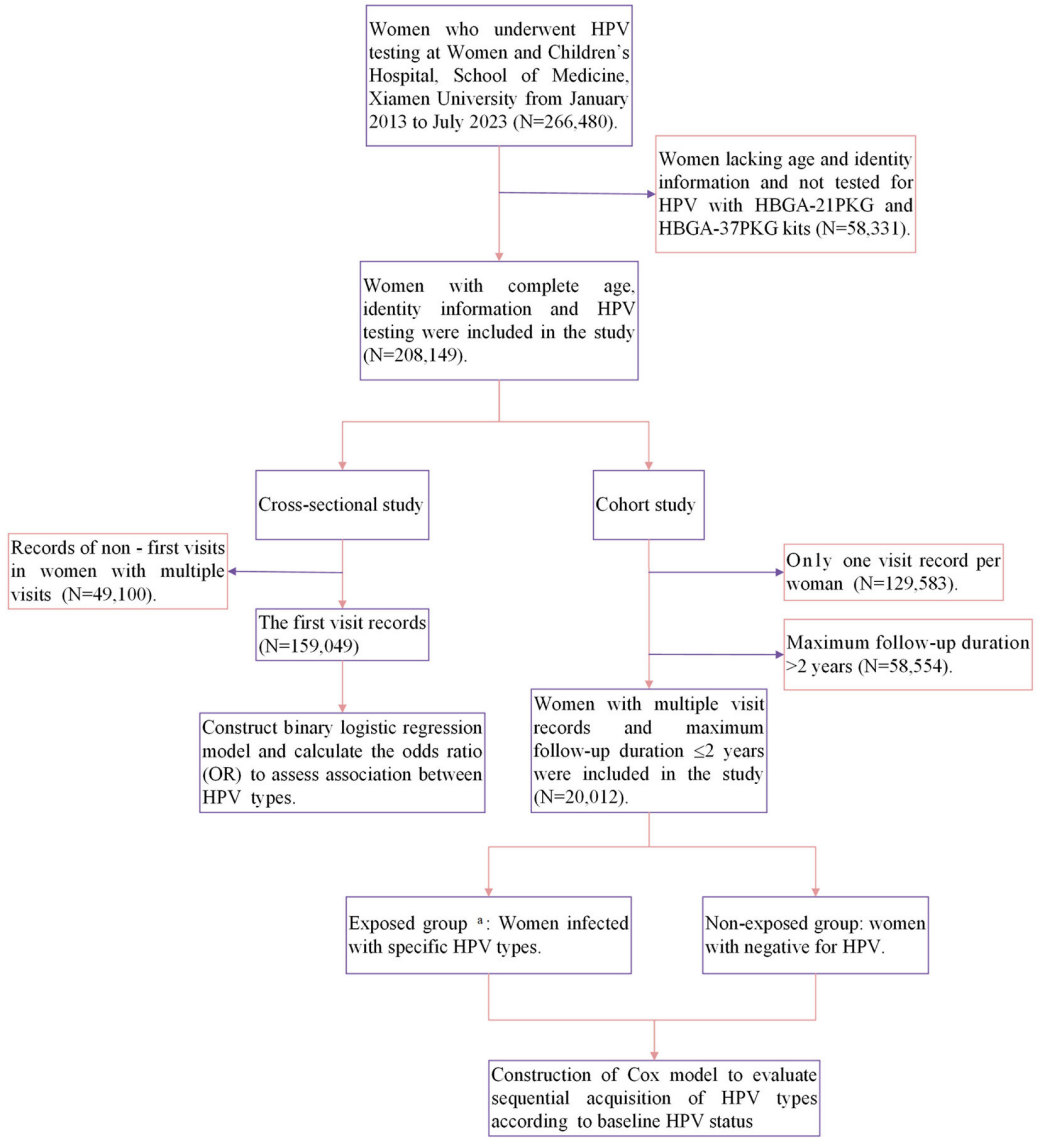
A flow diagram summarizing the study steps and data analysis procedures. a. Exposed group: baseline positivity for specific HPV (HPV-6/11/16/18/31/33/45/52/58/35/39/51/56/59/68).

Firstly, we included HPV genotyping result from the first visit for cross-sectional study (Figure 1). Difference in positive rate of HPV infection among different age, marital status and nationality groups were analyzed using χ^2^ test. Binary logistic regression was constructed to evaluated the association between infection with the HPV vaccine-targeted types (Models for HPV-6, −11, −16, −18, −31, −33, −45, −52, and−58) and infection with each of other types. Of note, all women were included in the analysis of common 21 types detected by 21 and 37 genotyping kits, while women presenting from 2018 to 2023 were further selected for the analysis of 16 additional types, HPV-26, −34, −40, −54, −55, −57, −61, −67, −69, −70, −71, −72, −73, −82, −83, and −84. HPV type-type interactions were assessed by calculating the odds ratios (OR) and 95% confidence intervals (CI) using a logistic regression model, with age as a covariate.

To further explore the association between HPV vaccine-targeted types and other types, we conducted an analysis of women with multiple visits (Figure 1). Due to the low prevalence of the newly added 16 HPV types, only the 21 types from 2013 to 2023 were included in the analysis. We compared the risk of sequential acquisition of non-vaccine-targeted HPV types between women infected with HPV vaccine-targeted types and women with negative for HPV. Considering that the majority of cervical HPV infection will clear within 2 years, the study allowed for a maximum follow-up duration of 2 years to assess acquisition (Wüstenhagen et al., 2018; Zhou et al., 2019) (if the subject was persistent infection, continue to observe for 2 years from the last detection of the HPV type). A sensitivity analysis was also conducted with an extended follow-up period of up to 3 years. Cox proportional hazards regression was used to estimate hazard ratios (HR) and 95% confidence intervals (CI) for analyses of interaction of HPV vaccine-targeted types and other HPV types. Women with vaccine-targeted types as the exposed group, and women with negative for HPV as the non-exposed group, HRs of <1.0 indicate that the risk of becoming infected with a specific non-vaccine-targeted HPV types was lower among those infected with a vaccine-targeted HPV types, thus implying potential type competition between these types.

In addition, we also analyzed the interactions between the remaining six high-risk HPV types (HPV-35, −39, −51, −56, −59, −68) and other types. The significance threshold was set at *P* < 0.05. Statistical analyses were performed using R version 4.3.2.

## 3 Results

### 3.1 Cross-sectional study

**Figure 2:**
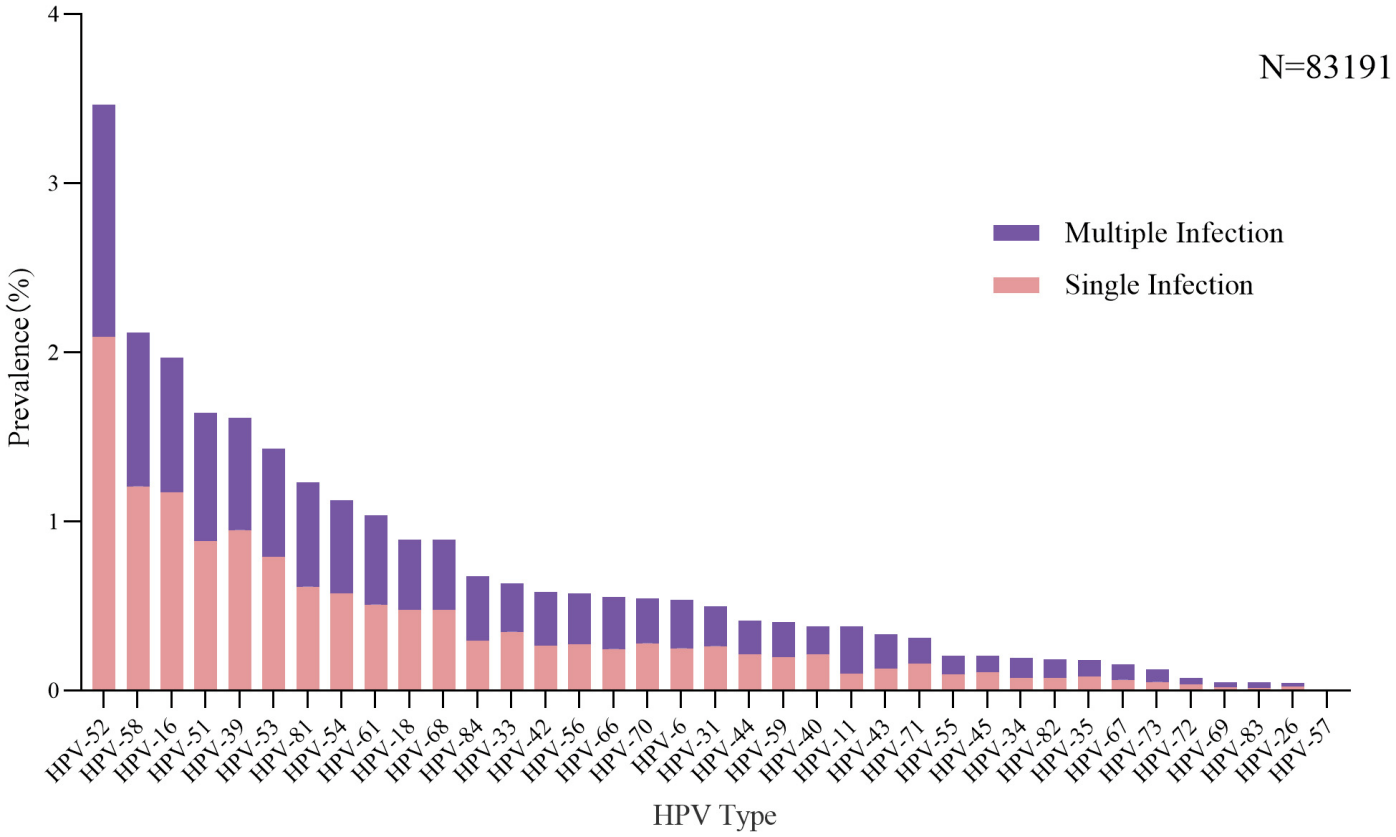
HPV types distribution of single and multiple infections. The colors **purple** and **pink** indicate single and multiple infections.

Among 159,049 women, with a median age of 35 (range, 15–92), 19.8% were positive for HPV (*n* = 31,502). The prevalence of HPV in women aged <35 years and women aged 35–65 years were 19.7% and 19.9%, respectively, and both lower than those aged >65 years (25.0%). The HPV infection rate is relatively higher in unmarried women (23.7%) compared to married women (17.8%), and women from minority nationalities (27.2%) have a higher HPV infection rate than Han women (19.7%) (*P* < 0.001). Single HPV type was detected in 74.3% (*n* = 23,419) and multiple HPV types were detected in 25.7% (*n* = 8,083) of positive population. Single HPV infection was the most common pattern across different age groups, marital statuses, and nationalities (Table 1). The analysis of HPV type distribution of single and multiple infections was conducted in women who underwent testing with the HBGA-37 PKG kits (*N* = 83191). As we have reported previously (Yao et al., 2024), HPV-52 (3.5%), −58 (2.1%), −16 (2.0%), −51 (1.6%), and−39 (1.6%) were the five most common types and were primarily single infections (Figure 2).

**Table 1:** Characteristics of participants at baseline.

	***N***	**All infection**			**Single infection**				**Multiple infection**			
		***n***	**Positive % (95%CI)**	***P***	***n***	**Positive % (95%CI)**	**Proportion % (95%CI)** ^a^	***P***	***n***	**Positive % (95%CI)**	**Proportion % (95%CI)** ^a^	***P***
**Total**	159,049	31,502	19.8 (19.6, 20.0)		23,419	14.7 (14.6,14.9)	74.3 (75.9,76.9)		8,083	5.1 (5.0,5.2)	25.7 (27.1,28.1)	
**Age**				<0.001				<0.001				<0.001
<35	87,483	17,223	19.7 (19.4,20.0)		12,550	14.3 (14.1,14.6)	72.9 (72.2,73.5)		4,673	5.3 (5.2,5.5)	27.1 (26.5,27.8)	
35–65	70,501	14,013	19.9 (19.6,20.2)		10,714	15.2 (14.9,15.5)	76.5 (75.8,77.2)		3,299	4.7 (4.5,4.8)	23.5 (22.8,24.2)	
>65	1,065	266	25.0 (22.4, 27.6)		155	14.6 (12.4,16.7)	58.3 (52.3,64.2)		111	10.4 (8.6,12.3)	41.7 (35.8,47.7)	
**Marital status**				<0.001				<0.001				<0.001
Unmarried	28,591	6,787	23.7 (23.2,24.2)		4,744	16.6 (16.2,17.0)	69.9 (68.8,71.0)		2,043	7.1 (6.8,7.4)	30.1 (29.0,31.2)	
Married	101,344	18,065	17.8 (17.6,18.1)		13,897	13.7 (13.5,13.9)	76.9 (76.3,77.5)		4,168	4.1 (4.0,4.2)	23.1 (22.5,23.7)	
Divorced, widowed	50	21	42.0 (28.3,55.7)		16	32.0 (19.1,44.9)	76.2 (58.0,94.4)		5	10.0 (1.7,18.3)	23.8 (5.6,42.0)	
Unknown	29,064	6,629	22.8 (22.3,23.3)		4,762	16.4 (16.0,16.8)	71.8 (70.8,72.9)		1,867	6.4 (6.1,6.7)	28.2 (27.1,29.2)	
**Nationality**				<0.001				<0.001				<0.001
Han	157,621	31,115	19.7 (19.5,19.9)		23,150	14.7 (14.5,14.9)	74.4 (73.9,74.9)		7,965	5.1 (4.9,5.2)	25.6 (25.1,26.1)	
Minority	1,422	387	27.2 (24.9,29.5)		269	18.9 (16.9,21.0)	69.5 (64.9,74.1)		118	8.3 (6.9,9.7)	30.5 (25.9,35.1)	
Unknown	6	0	0.0 (0.0,0.0)		0	0.0 (0.0,0.0)	0.0 (0.0,0.0)		0	0.0 (0.0,0.0)	0.0 (0.0,0.0)	

HPV, human papillomavirus; CI, confidence interval.

^*a*^The proportion of single or multiple infections among all HPV positive cases.

**Figure 3:**
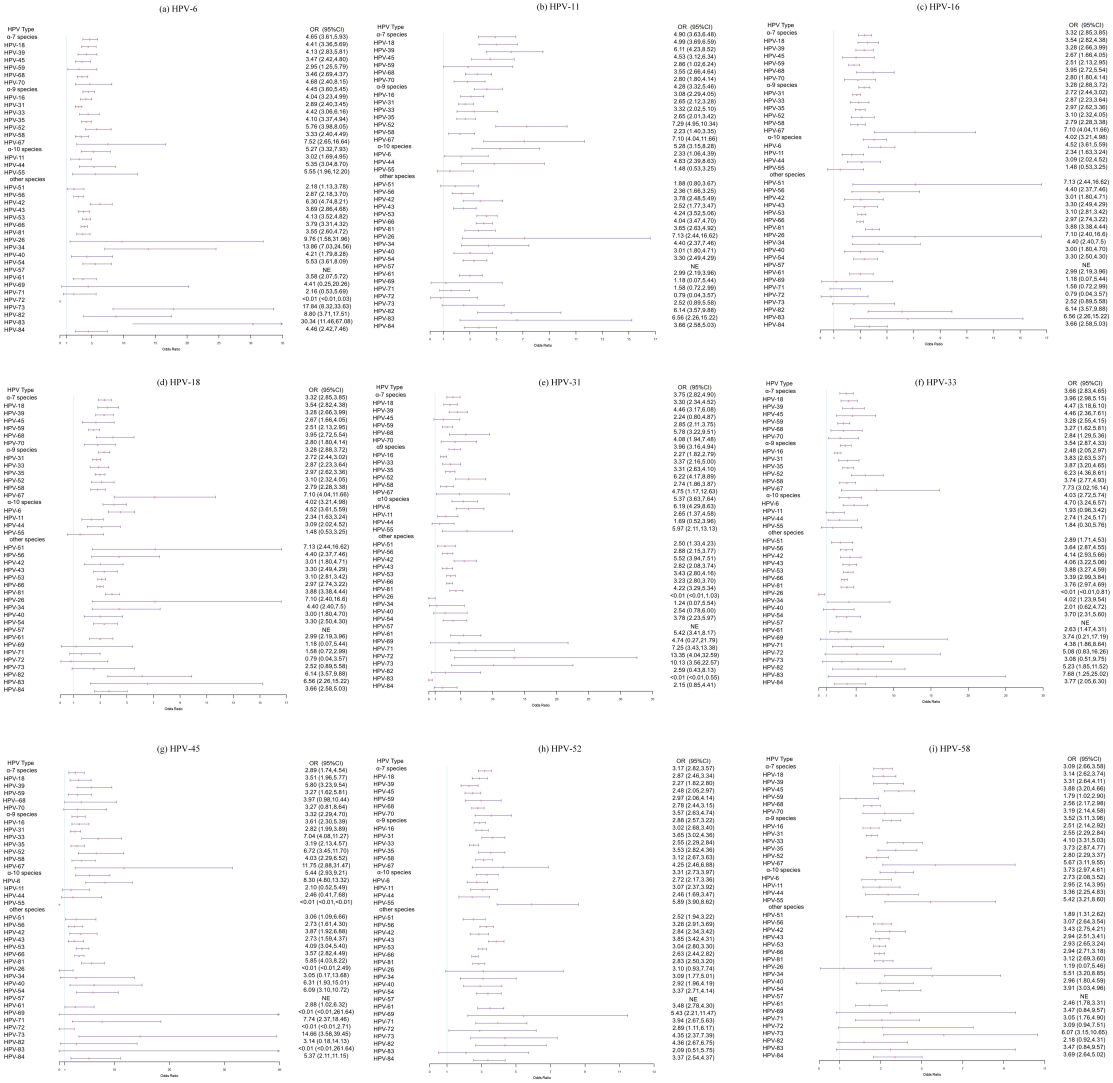
Age-adjusted odds ratios (ORs) for coinfections involving vaccine-targeted HPV types and other HPV types. **(a)** HPV-6 and other HPV types, **(b)** HPV-11 and other HPV types, **(c)** HPV-16 and other HPV types, **(d)** HPV-18 and other HPV types, **(e)** HPV-31 and other HPV types, **(f)** HPV-33 and other HPV types, **(g)** HPV-45 and other HPV types, **(h)** HPV-52 and other HPV types, and **(i)** HPV-58 and other HPV types.

As shown in Figure 3 and Supplementary Figure 1, in the pairwise comparisons of 15 HPV types (the current 9 vaccine-targeted types and other 6 high-risk types) and other 20 types, no significant negative association was observed. Further analysis of the association between these 15 types and the other 16 types introduced in 2018, statistically significant negative correlation were observed between several pairs of types: HPV-6 and HPV-72 (OR: < 0.01; 95%CI: < 0.01–0.03), HPV-18 and HPV-72 (OR: < 0.01; 95%CI: < 0.01–0.02), HPV-31 and HPV-83 (OR: < 0.01; 95%CI: < 0.01–0.55), HPV-33 and HPV-26 (OR: < 0.01; 95%CI: < 0.01–0.81), HPV-45 and HPV-55 (OR: < 0.01; 95%CI: < 0.01– < 0.01), HPV-56 and HPV-26 (OR: < 0.01; 95%CI: < 0.01–0.09), as well as HPV-59 and HPV-69 (OR: < 0.01; 95%CI: < 0.01–0.68).

### 3.2 Cohort study

Among women with type-specific negative for HPV at baseline, 1,348 women acquired any HPV infection during follow-up, with the incidence of 41.29/1,000 person-years, and the risk of acquisition of HR-HPV and LR-HPV were 32.51/1,000 person-years and 19.99/1,000 person-years, respectively. Similarly, HPV-52 was the most commonly acquired type, with the incidence of 8.39/1,000 person-years, followed by HPV-51, −58, −39 and−16, with the incidence of 5.98/1,000 person-years, 5.71/1,000 person-years, 4.88/1,000 person-years and 4.21/1,000 person-years, respectively. Most HPV types are prone to multiple infections, while HPV-53 is less likely to be detected in co-infections with other types (Table 2).

**Table 2:** Prevalence at enrollment and incidence of HPV infection in cohort of 20,038 women.

**HPV category**	**Prevalence at entry into study no. (%) of women**	**No. of participants**	**Follow-up. person-years**	**All incident infectious**		**no coinfections** ^**a**^		**One or more coinfections** ^**a**^	
				***n***	**incidence/1,000 person-years (95%CI)**	***n***	**incidence/1,000 person-years (95%CI)**	***n***	**incidence/1,000 person-years (95%CI)**
Any	10,062 (50.3)	9,950	32,645	1,348	41.29 (40.76,41.83)	1,001	30.66 (30.16,31.16)	347	10.63 (10.3,10.96)
**High risk**	8,340 (41.7)	18,443	37,225	1,210	32.51 (32.03,32.98)	770	20.69 (20.27,21.1)	440	11.82 (11.49,12.15)
HPV-16	1,565 (7.8)	18,444	55,825	235	4.21 (4.04,4.38)	92	1.65 (1.54,1.75)	143	2.56 (2.43,2.69)
HPV-18	658 (3.3)	19,697	58,528	186	3.18 (3.04,3.32)	80	1.37 (1.27,1.46)	89	1.52 (1.42,1.62)
HPV-31	388 (1.9)	19,624	59,128	81	1.37 (1.28,1.46)	28	0.47 (0.42,0.53)	53	0.90 (0.82,0.97)
HPV-33	488 (2.4)	19,924	58,957	113	1.92 (1.81,2.03)	52	0.88 (0.81,0.96)	61	1.03 (0.95,1.12)
HPV-35	112 (0.6)	19,900	59,987	48	0.80 (0.73,0.87)	23	0.38 (0.33,0.43)	25	0.42 (0.37,0.47)
HPV-39	916 (4.6)	19,096	58,005	283	4.88 (4.70,5.05)	139	2.40 (2.27,2.52)	144	2.48 (2.36,2.61)
HPV-45	128 (0.6)	19,884	59,928	50	0.83 (0.76,0.91)	19	0.32 (0.27,0.36)	31	0.52 (0.46,0.57)
HPV-51	979 (4.9)	19,033	57,878	346	5.98 (5.78,6.17)	135	2.33 (2.21,2.46)	211	3.65 (3.49,3.80)
HPV-52	2,286 (11.4)	17,726	53,654	450	8.39 (8.15,8.62)	201	3.75 (3.59,3.91)	249	4.64 (4.46,4.82)
HPV-56	373 (1.9)	19,639	59,263	151	2.55 (2.42,2.67)	48	0.81 (0.74,0.88)	103	1.74 (1.63,1.84)
HPV-58	1,497 (7.5)	18,515	55,894	319	5.71 (5.51,5.90)	147	2.63 (2.50,2.76)	172	3.08 (2.93,3.22)
HPV-59	288 (1.4)	19,724	59,560	93	1.56 (1.46,1.66)	24	0.40 (0.35,0.45)	69	1.16 (1.07,1.24)
HPV-68	554 (2.8)	19,458	58,820	169	2.87 (2.74,3.01)	65	1.11 (1.02,1.19)	104	1.77 (1.66,1.87)
**Low risk**	3,025 (15.1)	16,987	51,986	1,039	19.99 (19.64,20.33)	523	10.06 (9.8,10.32)	516	9.93 (9.67,10.18)
HPV-6	404 (2.0)	19,608	59,250	122	2.06 (1.94,2.17)	56	0.95 (0.87,1.02)	66	1.11 (1.03,1.20)
HPV-11	294 (1.5)	19,718	59,494	68	1.14 (1.06,1.23)	31	0.52 (0.46,0.58)	37	0.62 (0.56,0.69)
HPV-42	157 (0.8)	19,855	59,964	156	2.60 (2.47,2.73)	44	0.73 (0.67,0.80)	112	1.87 (1.76,1.98)
HPV-43	99 (0.5)	19,913	60,057	98	1.63 (1.53,1.73)	36	0.60 (0.54,0.66)	62	1.03 (0.95,1.11)
HPV-44	182 (0.9)	19,830	59,822	183	3.06 (2.92,3.20)	77	1.29 (1.20,1.38)	106	1.77 (1.67,1.88)
HPV-53	953 (4.8)	19,059	57,497	311	5.41 (5.22,5.59)	172	2.99 (2.85,3.13)	139	2.42 (2.29,2.54)
HPV-66	414 (2.1)	19,598	59,155	117	1.98 (1.87,2.09)	49	0.83 (0.76,0.90)	68	1.15 (1.06,1.24)
HPV-81	805 (4.0)	19,207	57,975	361	6.23 (6.03,6.42)	136	2.35 (2.22,2.47)	225	3.88 (3.72,4.04)
**Species** ^b^									
α-7 species	2,402 (12.0)	17,610	54,067	640	11.84 (11.56,12.11)	310	5.73 (5.54,5.93)	330	6.10 (5.9,6.31)
α-9 species	5,707 (28.5)	14,305	43,818	851	19.42 (19.05,19.79)	466	10.63 (10.35,10.92)	385	8.79 (8.52,9.05)
α-10 species	868 (4.3)	19,144	58,014	352	6.07 (5.87,6.26)	159	2.74 (2.61,2.87)	193	3.33 (3.18,3.47)

HPV, human papillomavirus; CI, confidence interval.

^*a*^Other HPV genotype detected at time of acquisition of the index infection.

^*b*^α-papillomavirus species: α-7 species comprises types 18, 39, 45, 59 and 68; α-9 species comprises types 16, 31, 33, 33, 35, 52, and 58; α-10 species comprises types 6, 11, and 44.

**Figure 4:**
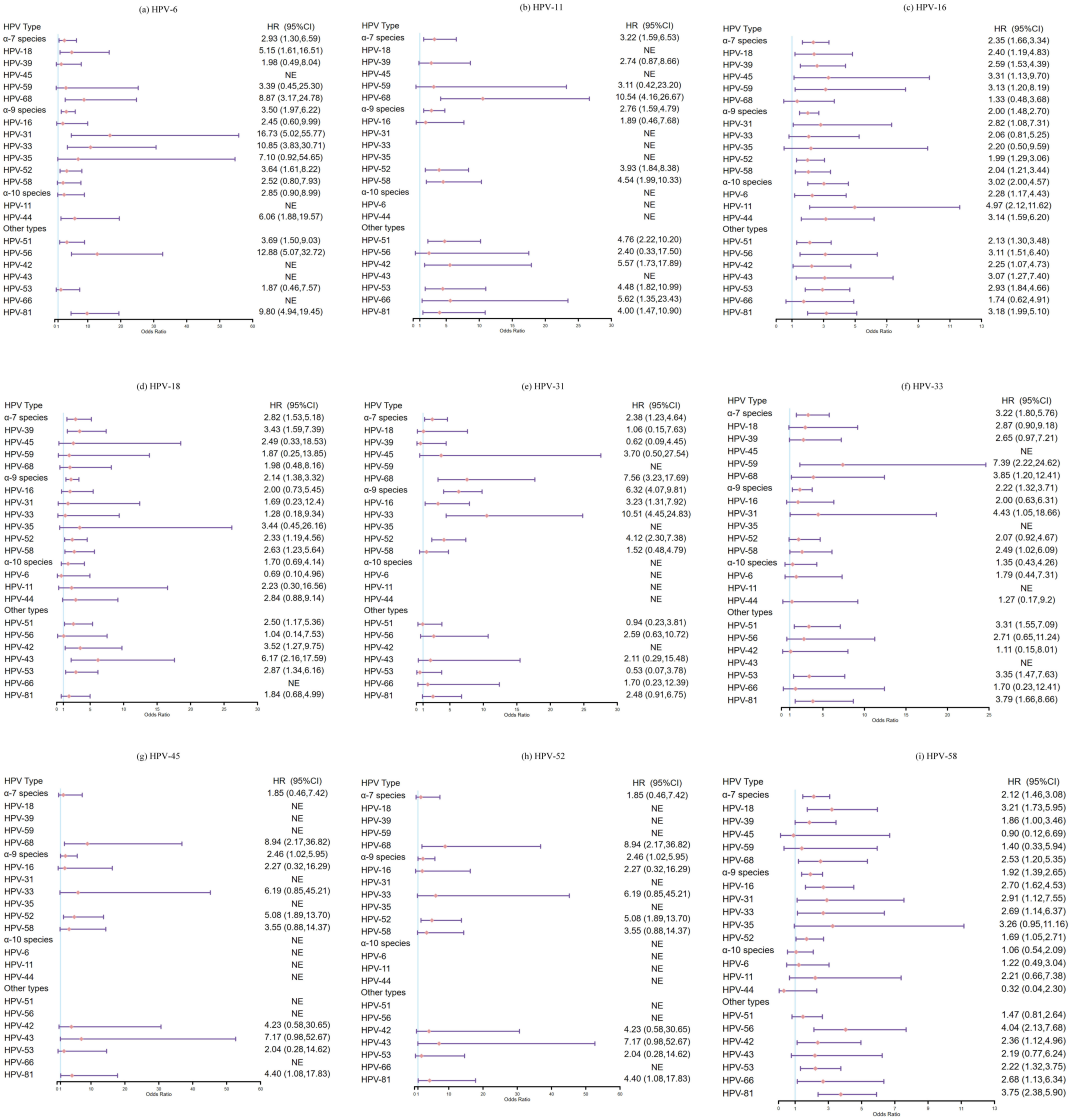
Hazard ratios and 95%CI for acquisition of non-vaccine-targeted HPV types in 2 years: women infected with HPV vaccine-targeted types (6, 11, 16, 18, 31, 33, 45, 52, and 58) vs. women with negative for HPV. **(a)** HPV-6 and other HPV types, **(b)** HPV-11 and other HPV types, **(c)** HPV-16 and other HPV types, **(d)** HPV-18 and other HPV types, **(e)** HPV-31 and other HPV types, **(f)** HPV-33 and other HPV types, **(g)** HPV-45 and other HPV types, **(h)** HPV-52 and other HPV types, and **(i)** HPV-58 and other HPV types.

In the subsequent analysis, women were grouped according to the presence of HPV infection, exposed group: women who were infected with 15 types, respectively, non-exposed group: women who were negative for HPV, and HPV type-specific incidence between two groups during follow-up period was compared. We found that prior infection with HPV-18 seemed to reduce the risk of acquiring HPV-6 (HR: 0.69, 95%CI: 0.10–4.96), however, the difference was not significant. Similar patterns were observed between HPV-31 and HPV-39 (HR: 0.62, 95%CI: 0.09–4.45), HPV-31 and HPV-53 (HR:0.53, 95%CI: 0.07–3.78), HPV-52 and HPV-6 (HR: 0.15, 95%CI: 0.02–1.05), HPV-58 and HPV-44 (HR: 0.32, 95%CI: 0.04–2.30), HPV-51 and HPV-6 (HR: 0.50, 95%CI: 0.07–3.58). Other results did not suggest that prior infection with any vaccine-targeted types (HPV-6, −11, −16, −18, −31, −33, −45, −52 and−58) inhibited acquisition of other types. Rather, the presence of preexisting infection with vaccine-targeted types actually increased the risk of acquiring other types (HR > 1.0, Figure 4 and Supplementary Figure 2).

To avoid underestimating the incidence of HPV infection, we further performed a sensitivity analysis by extending the follow-up period to 3 years. Although the differences were not statistically significant, we also found that prior infection with HPV-18 had reduced the risk of acquiring HPV-6 (HR: 0.70, 95%CI: 0.10–5.04), and similar trends were observed between HPV-31 and HPV-39 (HR: 0.59, 95%CI: 0.08–4.21), HPV-52 and HPV-6 (HR: 0.16, 95%CI: 0.02–1.17), HPV-58 and HPV-44 (HR: 0.58, 95%CI: 0.14–2.36), HPV-51 and HPV-44, −66 (HR: 0.62, 95%CI: 0.09–4.48; HR: 0.79, 95%CI: 0.11–5.71; respectively), HPV-68 and HPV-18(HR: 0.77, 95%CI: 0.11–5.51). In conclusion, no evidence was found that infection with HR-HPV types or HPV6/11 would reduce the risk of acquisition of other types (Supplementary Figures 3, 4).

## 4 Discussion

With the wide application of HPV vaccine, a concern has been raised that it may lead to HPV type replacement. It can provide insights concerning natural HPV type competition and potential for type replacement to assess pre-vaccine epidemiological data. In this study, we studied the possibility of HPV type competition using regression and cohort approach. We observed a large number of positive associations, while a few negative associations in HPV co-infection patterns among women from Xiamen, China in cross-sectional study, and we did not observe any statistically significant HR < 1.0, conversely, several statistically significant HR > 1.0 were observed in cohort study.

In general, our results were consistent with prior studies, which suggested null or positive associations between HPV types (Plummer et al., 2007; Vaccarella et al., 2010; Rositch et al., 2012; Vaccarella et al., 2013; Su et al., 2024). Two studies based on women undergoing cervical cancer screening at New Mexico and Shengjing Hospital of China Medical University, respectively, using logistic regression, reported significant negative associations between HPV-72 and HPV-84 (Yang et al., 2014) and between HPV-16 and HPV-52 (Nie et al., 2016). Consistent with study conducted by Elizabeth Louise Dickson (Dickson et al., 2013), negative association was also observed between HPV-31 and HPV-83 in our study. Moreover, negative associations were observed between HPV-6 and HPV-72, HPV-18 and HPV-72, HPV-33 and HPV-26, HPV-45 and HPV-55, HPV-56 and HPV-26, as well as HPV-59 and HPV-69, suggesting the possibility of type competition between these paired types. Although HPV-55, −69, −72 and−83 are considered as low-risk types, research shows that their risk is increasing (Brown et al., 1999; Kantathavorn et al., 2015; Shea et al., 2020; Gao et al., 2024). According to recent studies, HPV-26 was considered as high-risk types that may play a significant role in the development of cancer (Handisurya et al., 2007; Chen et al., 2018a,b; Zhou et al., 2019; Zhong et al., 2022). With the increasing coverage of HPV vaccine, infection with HPV-26, −72, −83 and −55 need to be monitored.

Few cohort studies were performed to explore HPV type competition. Previous natural history studies have not found that prior infection with one or more HPV types can inhibit infection with other types or promote the clearance of other types, but these studies did not specifically analyze vaccine-targeted types. Tota et al. (2016) conducted a cohort study to compare the incidence and clearance of over 30 non-vaccine-targeted types between the women who were positive for HPV vaccine-targeted types and women who were negative for HPV. The results showed that women infected with HPV vaccine-targeted types had a higher risk of infection with other types (HR > 1.0). Similarly, we also found that women infected with vaccine-targeted types had a higher risk of acquiring other types compared to uninfected women (HR > 1.0). Although not statistically significant, lower HPV incidence point estimates of HPV-6/18/39/44/66 were observed in women with prior HPV-18/31/51/52/58/68 infection vs. in women with negative for HPV. However, our cohort population consisted of patients who had visited the hospital multiple times, women with negative for HPV were less likely to follow up. Moreover, the number of incident infections (especially for less common HPV types) was low. Considering the differences of follow-up interval and follow-up frequency in the study population, and the confounding factors, such as sexual behavior, the finding needs further exploration in large sample population with regular follow-up cohort.

The strength of our study is its assessment of HPV type competition using regression and cohort approach simultaneously in a large sample size of Chinese women, which provide insights concerning natural HPV type competition and HPV type replacement. However, there are some limitations: (1) We did not collect high-risk behavioral factors for confounding analysis, such as the number of sexual partners, age of first sexual activity, smoking, and other details; (2) The limited sample size in the cohort follow-up population, especially the small number of HPV-negative women, may reduce the statistical power; (3) Only 21 types were included in the cohort study due to the small number of 37 types data. However, the types (HPV-26, −55, −69, −72, −83) showing negative associations in the cross-sectional analysis were the additional types detected in the 37-type assay, whereas these types were not analyzed in the cohort study. And further accumulation of 37 type data is required to corroborate the type competition between several pairs of HPV types in cohort studies.

In summary, the large sample cross-sectional study found that there is possibility of type competition between HPV-6 and HPV-72, HPV-18 and HPV-72, HPV-31 and HPV-83, HPV-33 and HPV-26, HPV-45 and HPV-55, HPV-56 and HPV-26 and HPV-59 and HPV-69. Future prospective studies are needed to assess the long-term potential for HPV type competition.

## Data Availability

The original contributions presented in the study are included in the article/Supplementary material, further inquiries can be directed to the corresponding author/s.
