# Supplementary material for: Epidemiologic evaluation of human papillomavirus type competition in unvaccinated women from Xiamen, China
Source: Front Microbiol. 2025 Apr 4;16:1546166. doi: 10.3389/fmicb.2025.1546166 (PMC12006131; doi:10.3389/fmicb.2025.1546166)
Supplement: Supplementary file 1 [file Data_Sheet_1.docx]

**Supplementar Figure 1. Age-adjusted odds ratios (ORs) for coinfections involving HR-HPV types and other HPV types.** (a): HPV-35 and other HPV types, (b): HPV-39 and other HPV types, (c): HPV-51 and other HPV types, (d): HPV-56 and other HPV types, (e): HPV-59 and other HPV types, (f): HPV-68 and other HPV types.

**Supplementary Figure 2. Hazard ratios and 95%CI for acquisition of non-targeted HPV types in 2 years: women infected with targeted HR-HPV types (35, 39, 51, 56, 59 and 68) versus women with negative for HPV.** (a): HPV-35 and other HPV types, (b): HPV-39 and other HPV types, (c): HPV-51 and other HPV types, (d): HPV-56 and other HPV types, (e): HPV-59 and other HPV types, (f): HPV-68and other HPV types.

**Supplementary Figure 3.** **Hazard ratios and 95% CI for acquisition of non-vaccine-targeted HPV types in 3 years: women infected with HPV vaccine-targeted types (6, 11, 16, 18, 31, 33, 45, 52, and 58) versus women with negative for HPV.** (a): HPV-6 and other HPV types, (b): HPV-11 and other HPV types, (c): HPV-16 and other HPV types, (d): HPV-18 and other HPV types, (e): HPV-31 and other HPV types, (f): HPV-33 and other HPV types, (g): HPV-45 and other HPV types, (h): HPV-52 and other HPV types, and (i): HPV-58 and other HPV types.

**Supplementary Figure 4: Hazard ratios and 95%CI for acquisition of non-targeted HPV types in 3 years: women infected with targeted HR-HPV types (35, 39, 51, 56, 59 and 68) versus women with negative for HPV.** (a): HPV-35 and other HPV types, (b): HPV-39 and other HPV types, (c): HPV-51 and other HPV types, (d): HPV-56 and other HPV types, (e): HPV-59 and other HPV types, (f): HPV-68and other HPV types.
